# Supporting rural primary care through Project ECHO: A brief case report

**DOI:** 10.1017/cts.2025.10196

**Published:** 2025-11-17

**Authors:** Jennifer L. Kraschnewski, Laura L. Felix, Sarah Cichy, Matthew Silvis, Chad Shaffer, Erik B. Lehman, Ruth Hogentogler, Cynthia H. Chuang

**Affiliations:** 1 Department of Medicine, Penn State College of Medicine, Hershey, PA, USA; 2 Department of Public Health Sciences, Penn State College of Medicine, Hershey, PA, USA; 3 Department of Family and Community Medicine, Penn State College of Medicine, Hershey, PA, USA; 4 ACMH Primary Care Center, New Bethlehem, PA, USA

**Keywords:** Rural health, primary care providers, Project ECHO, burnout, telementoring

## Abstract

Rural primary care providers report increasing rates of professional burnout, which can further exacerbate rural provider shortages and health disparities. From 2023 to 2025, the Project ECHO team at Penn State University developed and delivered an educational rural health telementoring program, collaboratively with stakeholders, to disseminate guideline-concordant care to rural primary care clinicians. The program focused on key rural topics and created a professional learning community aimed at decreasing participant burnout. Self-reported results of the pilot program’s participants (*n* = 106) demonstrate increased knowledge (*p* < .001) and reduced professional isolation. Future programing will expand data collection to explore longer-term impact.

## Introduction

Rural populations experience significant health disparities compared to their urban counterparts. Rural Americans face an increased incidence of chronic and preventable diseases including heart disease, cancer, stroke and respiratory disease and a reduced life expectancy [1,2]. Although multifactorial, a major driver of these disparities is access to care, including limited primary care physicians (PCPs). Although 17% of the US population lives in rural locations, only 10% of physicians practice there [3,4]. Rural areas have 5.1 PCPs and 11.1 advanced practice providers (APPs) – nurse practitioners, physician assistants, and certified nurse midwives – per 10,000 residents compared to 8.0 PCPs and 14.7 APPs in urban areas [5]. The rural health workforce shortage of over 20,000 PCPs in 2025 further contributes to disparities given that higher densities of PCPs are associated with lower mortality rates [3]. Current implemented solutions to alleviate the physician shortage include loan repayment programs, visa waivers, and scholarships. However, many programs focus on recruiting new physicians to areas with shortages rather than working to retain current rural physicians [6]. Therefore, identifying solutions to support rural PCPs and APPs (herein: rural primary care clinicians [RPCCs]) is critical to addressing rural health disparities.

Programs supporting rural health need to address the unique challenges faced by RPCCs, including higher patient ratios and longer hours, a wider range of conditions treated due to fewer sub-specialists, and professional isolation [3,7,8]. Project Extension for Community Healthcare Outcomes (ECHO) is an evidence-based, hub-and-spoke tele-education model aimed at improving the quality of care and reducing health disparities in rural communities [9–11]. A growing body of research demonstrates the efficacy and sustainability of the model across health care [12].

Our goal was to support RPCCs while disseminating guideline-concordant care to address rural health disparities. Therefore, we developed the Rural Health Project ECHO program, informed by rural health stakeholders and specifically designed to support RPCCs.

## Methods

### Project ECHO overview

The Rural Health Project ECHO program was developed over eight months in 2023 and delivered via two education cohorts, each consisting of nine, 60-minute sessions. The first cohort was held bi-weekly, November 3, 2023–March 8, 2024 with 32 participants, and the second cohort was held monthly, September 12, 2024–May 22, 2025, with 74 participants. Eight participants attended both cohorts. Session format followed the ECHO model® and included introductions, a 15-minute didactic presentation delivered by a medical expert on a rural health topic, 1–2 participant “spoke”-led de-identified patient case presentations, and a closing and debrief. Participant-shared de-identified patient case presentations served as the crux of each session ensuring mastery of content and skills. Following these case presentations, the participant group was encouraged to ask clarifying questions and offer recommendations. ECHO experts provided best-practice case recommendations, which were summarized verbally and by email follow-up. All sessions concluded with a hub-led debrief encouraging RPCCs to put into practice what they learned. The ECHO program was provided at no cost to participants, and all sessions were CME-eligible. Rurality was measured using the Center for Rural Pennsylvania’s county designations [13] (Table 1).

**Table 1.** Rural health ECHO participant demographics

Demographics	Cohort 1 participants *N* = 32 (%)	Cohort 2 participants *N* = 74 (%)	Total participants *N* = 106 (%)
**Reside or work in a rural PA county**			
Rural	18 (56.3)	30 (40.5)	48 (45.3)
Urban	12 (37.5)	40 (54.1)	52 (49.1)
NA	2 (6.3)	4 (5.4)	6 (5.7)
**Area of practice**			
Primary care	19 (59.8)	54 (73.0)	73 (68.9)
Specialty	5 (15.6)	8 (10.8)	13 (12.3)
Non-clinical care	8 (25.0)	12 (16.2)	20 (18.9)
**Occupation**			
Physician	7 (21.9)	32 (43.2)	39 (36.8)
Advanced practice provider	7 (21.9)	10 (13.5)	17 (16.0)
Nurse	11 (34.4)	13 (17.6)	24 (22.6)
Other	7 (21.9)	18 (24.3)	25 (23.6)
Not applicable/missing	0 (0.0)	1 (1.4)	1 (0.94)
**Self-reported burnout**			
I enjoy my work, I have no symptoms of burnout	5 (20.0)	22 (29.7)	27 (25.5)
Occasionally I am under stress and I do not always have as much energy as I once did, but I do not feel burned out	14 (56.0)	36 (48.7)	50 (47.2)
I am definitely burning out and have one or more symptoms of burnout, such as physical and emotional exhaustion	6 (24.0)	12 (16.2)	18 (17.0)
The symptoms of burnout that I am experiencing won’t go away. I think about frustration at work a lot	0 (0.0)	2 (2.7)	2 (1.9)
I feel completely burned out and often wonder if I can go on. I am at a point where I may need some changes or may need to seek some sort of help	0 (0.0)	2 (2.7)	2 (1.9)
Missing/NA	7 (21.9)	0 (0.0)	7 (6.6)

### Stakeholder-engaged curriculum development

During the planning phase, we utilized several approaches to ensure the highest-impact topics were identified and covered early in the curriculum. First, our expert rural health physician hub team provided feedback on proposed topics based on their personal experiences in rural health. Second, our Stakeholder Advisory Board, comprised of one rural PCP, one physician assistant employed at a rural health clinic, the CEO of a rural-based federally qualified health center, and a former Internal Medicine Chief at a hospital system serving rural communities, reviewed and provided feedback on the proposed curriculum. Lastly, during the online registration process, we included the proposed topic list for participants’ feedback.

Community-engaged approaches were used to develop the Rural Health Project ECHO program to ensure it would be supportive of RPCCs. First, we identified publicly available educational programs for RPCCs and toolkits to combat burnout [14]. Next, we met one-on-one with RPCCs and organizational leaders (*n* = 6) to discuss unique challenges and barriers faced when working in rural areas. Our stakeholders expressed the need for practical and timely information, with a less overt wellness focus, which was discussed to address burnout. This feedback led to a session structure that promoted a learning community for isolated providers while providing much-needed evidence-based medical knowledge. RPCC input during registration informed topic priority and selection. This resulted in curriculum refinement to nine meaningful sessions in each cohort (Table 2).

**Table 2.** Comparisons of mean knowledge retrospective pre–post ECHO session

Cohort	Session	Pre knowledge mean (95%) CI	Post knowledge mean (95% CI)	Difference (post–pre) mean (95% CI)	p-value
1	Rotator cuff evaluation (*n* = 12)	2.4 (2.0, 2.8)	3.2 (2.7, 3.6)	0.8 (0.3, 1.2)	0.002
1	Coronary artery disease diagnostic testing (*n* = 6)	2.7 (2.1, 3.2)	3.8 (3.3, 4.4)	1.2 (0.6, 1.8)	< 0.001
1	Obesity treatment with GLP-1^*^ agonists (*n* = 7)	2.7 (2.2, 3.3)	4.0 (3.5, 4.5)	1.3 (0.7, 1.9)	< 0.001
1	Treating opioid use disorder in the primary care setting (*n* = 6)	2.6 (2.1, 3.1)	3.6 (3.1, 4.1)	1.0 (0.4, 1.6)	0.003
1	Metabolic dysfunction-associated steatotic liver disease (*n* = 7)	2.3 (1.8, 2.8)	3.6 (3.1, 4.1)	1.3 (0.7, 1.9)	< 0.001
1	Parkinson’s and dementia: what providers need to know (*n* = 4)	2.6 (2.0, 3.2)	3.4 (2.7, 4.0)	0.8 (0.0, 1.5)	0.053
1	Hepatitis C (*n* = 3)	1.5 (0.8, 2.2)	3.5 (2.8, 4.2)	2.0 (1.1, 2.9)	< 0.001
1	Updates in contraception (*n* = 5)	3.0 (2.4, 3.6)	3.6 (3.0, 4.2)	0.6 (-0.1, 1.3)	0.081
1	Options for resistant depression (*n* = 3)	2.1 (1.4, 2.8)	3.4 (2.7, 4.1)	1.3 (0.5, 2.2)	0.004
2	Beyond statins for lipid lowering (*n* = 18)	2.7 (2.4, 3.0)	3.6 (3.3, 3.9)	0.8 (0.5, 1.2)	< 0.001
2	Advanced elements of the knee exam and options for injection therapies (*n* = 14)	2.6 (2.3, 2.9)	3.5 (3.2, 3.9)	0.9 (0.5, 1.3)	< 0.001
2	Diagnosing and initiating treatment for rheumatoid arthritis (*n* = 9)	2.3 (1.9, 2.7)	3.2 (2.8, 3.6)	0.9 (0.4, 1.4)	< 0.001
2	Nonmelanoma skin cancer for non-dermatologists (*n* = 6)	2.5 (2.0, 3.0)	3.2 (2.7, 3.7)	0.7 (0.1, 1.3)	0.028
2	Update on GLP-1^*^ agonist use for obesity, diabetes, and cardiovascular risk reduction (*n* = 22)	2.4 (2.1, 2.7)	3.4 (3.1, 3.7)	1.0 (0.7, 1.3)	< 0.001
2	Diagnosing and initiating treatment for bipolar disorder (*n* = 14)	2.7 (2.4, 3.0)	3.3 (3.0, 3.7)	0.6 (0.3, 1.0)	0.002
2	New congestive heart failure treatment guidelines (*n* = 11)	2.7 (2.4, 3.1)	3.5 (3.1, 3.8)	0.7 (0.3, 1.2)	0.001
2	Newer options for treating and preventing migraines (*n* = 10)	2.2 (1.9, 2.7)	3.3 (2.9, 3.7)	1.0 (0.5, 1.5)	< 0.001
2	Managing vasomotor symptoms of menopause (*n* = 10)	2.6 (2.2, 3.0)	3.6 (3.2, 4.0)	1.0 (0.5, 1.5)	< 0.001
	**Overall (*n* = 167)**	2.5 (2.4, 2.7)	3.5 (3.3, 3.6)	0.9 (0.8, 1.1)	< 0.001

Mean estimates, confidence limits, and p-values from a repeated measures model including terms for the session, pre/post time, and the interaction between the two terms. Means and confidence intervals were rounded to 1 decimal place.

* Glucagon-like peptide-1.

### Participant recruitment

Participant recruitment for Cohort 1 was conducted August–October 2023. A purchased list of RPCCs in select Pennsylvania counties and an internal database of Penn State’s Project ECHO alumni participants were used to send two separate email invitations. Penn State recruitment partners, including Primary Health Network, Pennsylvania Association of Community Health Centers (PACHC), and the Pennsylvania Area Health Education Center, were also engaged to distribute recruitment materials to their respective networks. Additionally, members of the hub team distributed recruitment materials to their networks of RPCCs. Finally, the ECHO team distributed flyers at the PACHC Annual Conference and Clinical Summit in October 2023. Cohort 2 recruitment was conducted June–August 2024 and replicated Cohort 1 recruitment methods, with the exception of conference flyers. While the ECHO program was designed with RPCCs in mind, enrollment was not restricted to PCPs and APPs. Participants included nurses, community health workers, and social workers involved in rural health care as well as rural clinic administrators (Table 1). Multidisciplinary teams from 11 clinics participated in the Rural Health Project ECHO program. Registrants from non-rural counties were not excluded from participation as areas within these counties may be classified as rural using other definitions of rurality.

### Program evaluation

Participant evaluation data was collected at registration (baseline), after each ECHO session (post-session), and at the end of the ECHO program (post-program). At registration, participants completed an electronic survey collecting demographic information and pre-ECHO measurements. Burnout was self-reported by participants pre- and post-participation using the non-proprietary single item burnout measure [15]. This measure instructs participants to rate their level of burnout based on a 5-point ordinal scale, where 1 = “I enjoy my work. I have no symptoms of burnout.” and 5 = “I feel completely burned out and often wonder if I can go on. I am at the point where I may need some changes or may need to seek some sort of help.” Study data were collected and managed using REDCap electronic data capture tools hosted at Penn State Health Milton S. Hershey Medical Center and Penn State College of Medicine [16].

Participants were evaluated after each ECHO session (nine sessions per cohort) regarding their medical knowledge on each curriculum topic. Participant knowledge changes were self-reported using a 5-point ordinal scale, using two separate questions phrased, “Please rate your knowledge before/after the ECHO session.” To minimize recall bias, the knowledge questions were asked using a retrospective post-pre approach, with the questions appearing in the session evaluation survey after each ECHO session. A generalized linear model for repeated measures, which included predictors for session (1–9), time within session (pre, post), and their interaction, was used to compare differences in mean knowledge between pre- and post-timepoints within each of the nine ECHO sessions and collectively across the program. The model used mean change as the measure of central tendency between pre- and post-session, and the distribution of responses was examined using histograms and residual diagnostics. Model assumptions were assessed using plots of the standardized and studentized residuals from the model as well as box plots of the outcome variable itself. All comparison testing was completed using SAS 9.4 software [17]. Also assessed were participants’ ability to provide care, intent to make changes in their practice due to ECHO session participation, and sense of professional isolation. These questions were developed using Moore’s Levels for CME framework [18].

## Results

Two cohorts of the pilot Rural Health Project ECHO program have been completed with 106 participants (98 unique). Table 1 shows key participant demographic breakdowns for each cohort and overall. Approximately two-thirds of participants worked in primary care, and nearly half (45.3%) reported residing or working in rural counties in Pennsylvania. Professional roles held by all participants include physicians (36.8%), APPs (16.0%), nurses (22.6%), and other professionals involved in rural healthcare (23.6%), including community health workers, social workers, and rural clinic administrators.

A total of 167 ECHO session evaluations were collected for the 18 ECHO sessions across cohorts 1 (19 unique respondents) and 2 (46 unique respondents). Most participants (*n* = 65, 61.3%) completed at least one post-session evaluation. Participants self-reported changes in knowledge related to each session topic. The repeated measures model analysis of mean knowledge by session (Table 2) showed statistically significant (*p* < 0.05) increases in mean knowledge for 16 out of the 18 sessions. Mean knowledge was also assessed overall for all 18 sessions and was found to be statistically significant (*p* < .001)

Participants were also asked about changes in their ability to provide care, intent to make changes in their practice, and sense of professional isolation (Table 3). Among participants completing post-session evaluations (*n* = 65), many agreed or strongly agreed that because of their participation in an ECHO session, their sense of professional isolation decreased (71.9%), their ability to provide care in the topic area improved (88.6%), and they intended to make changes in their practice (74.3%).

**Table 3.** Aggregated session evaluation results, cohorts 1 and 2 (*n* = 167)

Competency	Strongly agree	Agree	Neutral	Disagree	Strongly disagree
Decreased sense of isolation	20	100	39	8	0
Improved ability to provide care	30	118	18	0	1
Intend to make changes in practice	26	98	36	5	2

## Discussion

This pilot program found that participation in the Rural Health ECHO series can improve provider knowledge on a range of medical topics important to RPCCs, supporting their treatment of a wider range of conditions. This could potentially lead to a decrease in specialty referrals, alleviating pressure on the healthcare system and lessening patient burden. Programs such as this one could be used to enhance knowledge of evidence-based topics to address rural health disparities as well as prevent or alleviate professional isolation in RPCCs by establishing supportive virtual peer learning communities. While this program was initially conceptualized to address rural provider burnout, baseline participant data did not demonstrate high levels of burnout. It is possible that providers experiencing burnout are less likely to seek recurring continuing education sessions.

Limitations to our findings include a limited post-evaluation sample size. While all participants completed the baseline survey, the post-program response rate was 12.5% in Cohort 1 and 9.5% in Cohort 2. Due to the small post-program response rate and low pre-burnout values, post-program self-reported burnout values were not reported or analyzed. Another potential limitation was our use of the Center for Rural Pennsylvania’s definition for rural counties, which may have missed some rural providers. There are many definitions used to distinguish rural areas which may capture urban-designated counties with rural providers, such as (1) the US Bureau of the Census’ urban classification, (2) the Office of Management and Budget’s definition of metropolitan and non-metropolitan areas, (3) the Federal Office of Rural Health Policy’s Rural Urban Commuting Area Codes, and (4) the Health Resources and Services Administration’s definition of rural areas.

This study’s strength is the significant knowledge gain from ECHO attendance, supporting evidence of the model’s effectiveness in provider education [12]. Further, this program was strengthened by the stakeholder-focused curriculum design and range of participant roles in rural healthcare.

Future plans for this program include two additional cohorts 2026–2027, with the addition of a one-day in-person gathering to accompany the ECHO education sessions. This new event will provide RPCCs expert-led, hands-on training and networking with ECHO colleagues, specialists, and medical students and resident physicians training to work in rural settings. Future cohorts will incorporate strategies to increase post-program evaluation response rates.

## Conclusions

Project ECHO presents many opportunities for future rural health programing. The pilot Rural Health ECHO program demonstrates the feasibility of the ECHO model and the viability of engaging rural health stakeholders in planning curriculum to ensure participants receive education tailored to address rural health challenges.
